# The Combined Effect of High Ambient Temperature and Antihypertensive Treatment on Renal Function in Hospitalized Elderly Patients

**DOI:** 10.1371/journal.pone.0168504

**Published:** 2016-12-19

**Authors:** Iftach Sagy, Alina Vodonos, Victor Novack, Boris Rogachev, Yosef S. Haviv, Leonid Barski

**Affiliations:** 1 Clinical Research Center, Soroka University Medical Center, Beer-Sheva, Israel; 2 Internal Medicine Division, Soroka University Medical Center, Beer-Sheva, Israel; 3 Faculty of Health Sciences, Ben-Gurion University of the Negev, Beer-Sheva, Israel; 4 Department of Nephrology, Soroka University Medical Center, Beer-Sheva, Israel; Medizinische Universitat Graz, AUSTRIA

## Abstract

**Background:**

The aging kidney manifests structural, functional as well as pharmacological changes, rendering elderly patients more susceptible to adverse environmental influences on their health, dehydration in particular.

**Hypothesis:**

Higher temperature is associated with renal function impairment in patients 65 years and older who routinely take thiazide and/or ACE-inhibitors/ARBs.

**Methods:**

We obtained health data of patients older than 65 who were admitted to a large tertiary center during the years 2006–2011, with a previous diagnosis of hypertension, and treated with thiazide, ACE-inhibitors/ARBs or both. We collected environmental data of daily temperature, available from collaborative public and governmental institutions. In order to estimate the effect of daily temperature on renal function we performed linear mixed models, separately for each treatment group and creatinine change during hospital admission.

**Results:**

We identified 26,286 admissions for 14, 268 patients with a mean age of 75.6 (±6.9) years, of whom 53.6% were men. Increment in daily temperature on admission of 5°C had significant effect on creatinine increase in the no treatment (baseline creatinine adjusted 0.824 mg/dL, % change 1.212, % change 95% C.I 0.082–2.354) and dual treatment groups (baseline creatinine adjusted 1.032mg/dL, % change 3.440, % change 95% C.I 1.227–5.700). Sub-analysis stratified by advanced age, chronic kidney disease and primary diagnosis on hospital admission, revealed a significant association within patients admitted due to acute infection and treated with dual therapy.

**Conclusion:**

Whereas previous studies analyzed sporadic climate effects during heat waves and/or excluded older population taking anti-hypertensive medications, the present study is novel by showing a durable association of temperature and decreased renal function specifically in elderly patients taking anti-hypertensive medications.

## Introduction

Elderly people (over 65 years old) are more susceptible to environmental influences on their health including dehydration [1]. Since aging is associated with several physiological and pathological processes, advanced age is associated with renal function impairment [2]. Acute kidney injury among the elderly is an important risk factor for further deterioration of their kidney function, possibly culminating to end stage kidney disease[3].

The use of antihypertensive medications, commonly prescribed for elderly patients, may augment the risk for insensible loss, dehydration and eventually impaired kidney functions in several mechanisms. [4,5]. Previous studies have found an association between high ambient temperature (specifically during extreme heat waves) and elderly health outcomes, e.g. increased mortality rates, ED visits and hospital admissions [6,7]. Thus, elderly patients are more vulnerable to dehydration and insensible loss, especially during high temperature periods even when they stay indoors [8].

In this study we sought to explore the effect of ambient temperature on renal function in hypertensive elderly patients taking medications with potential effect on kidney function, hypothesizing that higher temperature will be associated with renal function impairment especially in patients treated with common antihypertensive drugs.

## Methods

### Study population

We obtained health data of all patients above 65 years old admitted to Soroka University Medical Center (SUMC) during the years 2006–2011. SUMC is the only medical center in this area serving a population of approximately 700,000 as the only primary hospital in Northern Negev desert and nearly 1.2 million as a tertiary hospital. This study was approved by the institutional ethics committee of Soroka University Medical Center. The institutional ethics committee waived the need for written informed consent from the participants due to the anonymously data analysis. We extracted demographic and clinical data from the records computerized database. We used the first measure of laboratory and other clinical parameters (e.g. blood pressure) of each patient, taken during the first hour from the admission to SUMC emergency department (ED). The primary diagnosis for admission was extracted from the ED medical records. Past medical history was extracted from previous hospitalizations or outpatient clinics encounters according to ICD-9 codes and definitions. All variables had less than 5% of missing values, except for the chronic kidney disease stage (See below). Patients were identified by the national ID number which was encoded before data analysis.

### Increase in serum creatinine

Change in serum creatinine, rather than estimated glomerular filtration rate (eGFR) was selected to reflect change in renal function. In acute kidney injury (AKI) the correlation between the GFR and serum creatinine is poor. Therefore, KDIGO guidelines for AKI focus on serum creatinine changes and urine output rather than eGFR[9]. We used the first serum creatinine that was measured on ED admission to reflect a prior effect of the ambient temperature on renal function. We calculated the expected first creatinine change vs. the mean daily temperature on the day of admission. In addition, the degree of serum creatinine change in the index hospitalization was calculated for each patient vs. the corresponding mean outpatient serum creatinine in the year preceding the index hospitalization (available for 16,164admissions61.5%). This method had been validated by a panel of nephrologists to be a proxy of the baseline kidney function prior to admission [10]. Patients on renal replacement therapy or GFR below 15 mm/min on admission were excluded from the study. Because of the epidemiologic nature of our study, even statistical changes in serum creatinine were absolutely very small and did not reach the >0.3 mg% serum creatinine rise as defined for AKI by KDIGO.

### Medications

We divided the anti-hypertensive drugs (consumed at least three month prior the hospitalization) into three main groups: angiotensin-converting-enzyme inhibitors (ACE) or angiotensin receptor blockers (ARBs) without thiazide (1) thiazide only and (2) ACE-inhibitors or ARBs with thiazide (3). Patients with none of the aforementioned medications comprised the reference group.

### Meteorological Data

The environmental data were available from collaborative public and governmental institutions [11]. Spatial historical data included meteorological parameters (maximum, minimum, average of daily temperature and relative humidity). The climate in the Negev desert is hot and dry, featured by a negligible amount of rainfall through the entire year. The average temperature in the summer season during our study was 26.9°C (±1.9°C) compared to 14.1°C (±3.4°C) during the winter. Seasons were defined according to Alpert et al.[12]: winter (December 7-March 30), spring (March 31-May 30), summer (May 31-September 22) and autumn (September 23-December 6).

### Statistical analysis

Data are expressed as mean ± standard deviation (SD) or number and percentage. To compare the patient characteristics according to the drug groups we used analysis of variance (ANOVA) and chi-square tests.To estimate the effect of the daily outdoor temperature on serum creatinine, log transformed creatinine were modelled using mixed linear models, with a random effect for each patient and fixed effect of daily temperature age, gender, mean arterial blood pressure, congestive heart failure (CHF), peripheral vascular disease (PVD), diabetes mellitus, cerebro-vascular accident (CVA),dementia and chronic liver disease separately for each treatment group. Coefficients were antilog transformed to the original units, and results are presented as percent change in creatinine and 95% confidence intervals (CI).We used mixed linear models to control for cluster effect of repeated hospitalizations of the same patient as mentioned, since the unit of evaluation was hospitalization.We excluded patients with baseline estimated GFR≤ 15 mL/min/1.73 m^2^ (chronic kidney disease stage V) due to the poor renal compensatory capability and high prevalence of renal replacement therapy [13]. To examine the factors associated with change in creatinine (creatinine levels during admission compared to baseline creatinine) we performed a linear mix model with delta in creatinine levels as a dependent variable. We calculated the expected increase in the creatinine for each individual characteristic beyond the effect of the outdoor temperature (stratified by age, infection on admission and drug group). Log transformed creatinine change vs. spline of daily mean temperature figure confirmed the linear association of these variables. All of the above models were fitted using maximum likelihood methods and were selected based on the criteria of minimizing Akaike’s Information Criterion [14]. In addition, models were built upon statistical significance (α<0.05) of the univariate analysis, model parsimony and clinical relevance [15]. Data analysis was performed using SPSS version 22 (SPSS Inc. Chicago, Illinois).

## Results

### Patient characteristics

**Fig 1:**
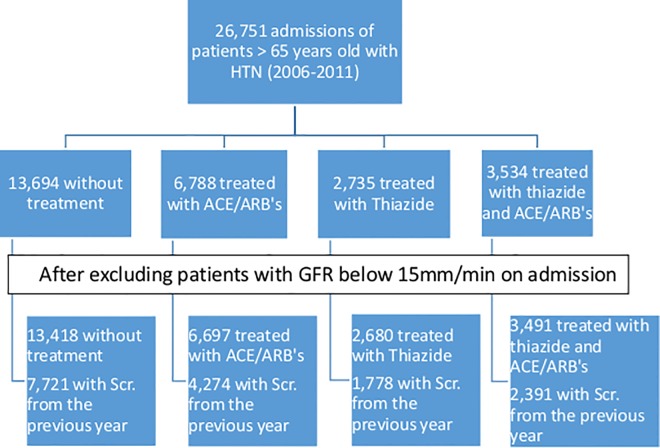
Study flow chart of four medication groups.

We identified 26,751 admissions of 14, 605 patients during the study period (2006–2011). After excluding patients with GFR < 15 mL/min/1.73 m^2^ there were 26,286 admissions which were included in the final analysis (Fig 1). Table 1 presents the baseline characteristics of patients according to the type of medication. The average patient age was 75.8 (±6.8) years, and 54.1% were men. Among them, 281 (1.1%) patients had systolic blood pressure below 90 on admission, and 270 (1.8%) died during the index hospitalization. Patients treated with either a thiazide or a thiazide/ACE/ARBs combination had higher rates of CHFcompared to patients treated with only ACE/ARBs or patients treated with none of the study medications (17.8%, 19.1% vs. 4.6%, 5.0%, respectively).

**Table 1:** Baseline demographic and clinical characteristics of patients according to type of treatment on hospital admission.

		None(n = 13,418)	ACE/ARBs (1)(n = 6,697)	thiazide (2)(n = 2,680)	ACE/ARBs+ thiazide (3)(n = 3,491)	P value
Demographic Characteristics	Male gender, n(%)	7,431 (55.4)	3,665 (54.7)	1,306 (48.7)	1,806 (51.2)	<0.001
	Age (years), mean ±SD	75.7 (±6.8)	75.5 (±6.7)	76.5 (±6.8)	76.2 (±6.7)	<0.001
**Blood pressure on admission**	Systolic blood pressure(mmHg), mean ±SD	138.9 (±51.9)	141.9 (±42.3)	137.6 (±38.9)	141.5 (±52.8)	<0.001
	Diastolic blood pressure (mmHg), mean ±SD	74.9 (±21.9)	76.1 (±29.7)	74.2 (±19.3)	74.3 (±19.5)	<0.001
	Mean arterial blood pressure (mmHg), mean ±SD	96.2 (±22.8)	98.0 (±25.2)	95.3 (±19.6)	96.7 (±22.4)	<0.001
**Co-morbid conditions**	NewMyocardial infarction, n(%)	811 (6.0%)	433 (6.5%)	148 (5.5%)	192 (5.5%)	0.155
	Heart failure,n(%)	672 (5.0%)	308 (4.6%)	511 (19.1%)	620 (17.8%)	<0.001
	Diabetes Mellitus, n(%)	3,647 (27.2)	2,582 (38.7)	899 (33.6)	1,371 (39.4)	<0.001
	Peripheral vascular disease, n(%)	350 (2.6%)	177 (2.6%)	88 (3.3%)	122 (3.5%)	0.012
	Stroke, n(%)	827 (6.2%)	565 (8.5%)	187 (7.0%)	280 (8.0%)	<0.001
	Liver disease, n(%)	307 (2.3%)	48 (0.7%)	100 (3.7%)	27 (0.8%)	<0.001
	Dementia, n(%)	571 (4.3%)	202(3.0%)	75 (2.8%)	74 (2.1%)	<0.001
	Cancer, n(%)	223 (1.7%)	104 (1.6%)	41 (1.5%)	45 (1.3%)	0.450
**Primary diagnosis for admission**	Infection, n(%)	667 (5.0%)	290 (4.3%)	94 (3.5%)	131 (3.8%)	<0.001
	Cardiovascular, n(%)	5595 (41.7%)	3227 (48.2%)	1140 (42.5%)	1679 (48.1%)	<0.001
	Respiratory, n(%)	2652 (19.8%)	1199 (17.9%)	622 (23.2%)	720 (20.6%)	<0.001
	Gastrointestinal, n(%)	1271 (9.5%)	501(7.5%)	244(9.1%)	216 (6.2%)	<0.001
	Renal or dehydration, n(%)	492 (3.7%)	219 (3.3%)	102 (3.8%)	135 (3.9%)	0.346
	Other, n(%)	2741 (20.4%)	1261 (18.8%)	478 (17.8%)	610 (17.5%)	<0.001

ACE/ARBs—angiotensin-converting-enzyme inhibitor or angiotensin receptor blockers

Mean arterial blood pressure was higher on admission in patients taking ACE/ARBs alone vs. other groups and a previous history of stroke was more prevalent in patients taking ACE/ARBs, either alone or in combination with a thiazide, possibly reflecting confounding by indication. Similarly, higher rates of dementia were observed in patients not receiving ACE/ARBs or thiazide and higher rates of cardiovascular events as primary admission diagnosis were observed in patients on ACE/ARBs (Table 1). Of special note, there was no difference in the rate of "renal or dehydration" primary admission diagnosis among the medication treatment groups (Table 1). Baseline laboratory characteristics from ED admission of the study cohort is shown in Table 2. While laboratory values were extracted for the entire cohort, only 16,164 (61.5%) patients had creatinine measurements from the previous year, allowing calculation of the CKD stage. Baseline CKD was inversely related to the use of ACE/ARBs.

**Table 2:** Baseline laboratory characteristics of patients according to type of treatment on hospital admission.

	None(n = 13,418)	ACE/ARBs (1) (n = 6,697)	thiazide (2) (n = 2,680)	ACE/ARBs+ thiazide (3) (n = 3,491)	P value
First creatinine (mg/dL) mean ±SD	1.02±0.45	1.02±0.39	1.21±0.56	1.18±0.49	<0.001
Maximum Creatinine (mg/dL) mean ±SD	1.13±0.58	1.12±0.50	1.37±0.76	1.33±0.65	<0.001
Last Creatinine (mg/dL) mean ±SD	1.02±0.48	1.02±0.41	1.23±0.61	1.18±0.53	<0.001
Estimated GFR on admission (mL/min), mean ±SD	80.7 (±24.7)	82.2 (±23.2)	72.1 (±27.2)	75.0 (±25.5)	<0.001
Urea (mg/dL) mean ±SD	47.7 (±26.3)	47.4 (±21.3)	62.7 (±38.2)	60.5 (±33.2)	<0.001
Sodium (mEq/L), mean ±SD	138.3 (±3.6)	138.3 (±3.6)	137.9 (±4.1)	138.0 (±3.8)	<0.001
Uric Acid (mg/dL) mean ±SD	5.9 (±2.0)	6.0 (±1.9)	7.3 (±2.5)	7.2 (±2.2)	<0.001
Albumin (g/dl), mean ±SD	3.7 (±0.5)	3.8 (±0.5)	3.7 (±0.5)	3.7 (±0.5)	<0.001
Stage 1 or no CKD*	4442(53.6%)	2480 (54.2%)	769 (45.7%)	1119 (45.7%)	<0.001
CKD Stage 2*	2557 (30.9%)	1470 (32.1%)	573 (34.1%)	829 (35.2%)	
CKD Stage 3	996 (12.0%)	499 (10.9%)	277 (16.5%)	352 (14.9%)	
CKD Stage 4*	207 (2.5%)	95 (2.1%)	54 (3.2%)	41 (1.7%)	
CKD Stage 5*	83 (1.0%)	33 (0.7%)	8 (0.5%)	16 (0.7%)	

*Based on the mean creatinine in the preceding year prior to the admission which was available for 61.5% of study cohort

ACE/ARBs—angiotensin-converting-enzyme inhibitor or angiotensin receptor blockers, GFR–glomerular filtration rate

### Association of temperature increment with increase in creatinine

Results of the mixed linear models for the effect of temperature on creatinine are presented in Table 3. Increment of 5 degrees (C°) was significantly associated with creatinine rise among patients in the non-treatment group and in group 3 (ACE/ARBs+ thiazide), (% change in creatinine = 1.21% and 3.44%, p value = 0.036 and 0.002 respectively), compared to non significant change among patients in group 2 (thiazide) and groups 3 (ACE/ARBs). Intriguingly, the association of creatinine and daily mean temperature showed co-linearity in all treatment groups. Stratified analysis by seasons (summer, autumn, winter and spring in S1 Fig), did not show association with creatinine increment.

**Table 3:** Baseline creatinine and estimates of the effect of daily temperature on renal function, by medication groups*.

ALL SEASONS		Increase of5°C			
Treatment groups	Baseline Creatinine (ml/min)	% change in Creatinine	95% CI for % change in Creatinine		P value
None	0.824	1.212	0.082	2.354	0.036
ACE/ARBs	0.895	1.147	-0.218	2.530	0.100
thiazide	1.006	0.702	-1.872	3.345	0.597
ACE/ARBs and thiazide	1.032	3.440	1.227	5.700	0.002

*Adjusted for mean arterial blood pressure, congestive heart failure, peripheral vascular disease, diabetes mellitus, dementia, cerebrovascular accident, chronic liver disease, Age and Gender

ACE/ARBs—angiotensin-converting-enzyme inhibitor or angiotensin receptor blockers

To further investigate the effect of ambient temperature on AKI, we conducted an analysis of AKI incidence according to the 2007 AKIN criteria. In our cohort 16,194 patients who had at least one measurement of serum creatinine from the preceding year, had also met one AKIN criterion for AKI stage 1 (abrupt rise in creatinine of more than 150% but less than 200% or more than 0.3mg/dL from baseline). Since urine output measure is not reliable without urinary catheter, we did not evaluate the change in urine output criterion. The results of logistic regression for developing AKI, show a significant association of AKI and 5°C increment in elderly patients taking either thiazide alone or a combination of thiazide and ACE/ARBs. Additionally, several subgroups were at increased risk to develop AKI, such as males, and patients with CHF, diabetes, cerebrovascular accident and peripheral vascular disease (S1 Table).

**Fig 2:**
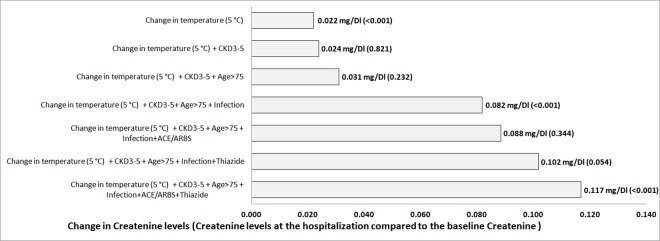
The cumulative increase in serum creatinine (mg/dL) for the addition of each clinical characteristic. The additional p value for each parameter is presented in brackets. ACE/ARBs—angiotensin-converting-enzyme inhibitor or angiotensin receptor blockers, CKD–chronic kidney disease

Table 4 shows a significant association between 5°C increment and creatinine increase relative to baseline AKI. Creatinine change was also found significant among patients with a primary diagnosis of fever or sepsis and taking both thiazide + ACE/ARBs group. We did not find a significant association for patients with other primary diagnosis on hospital admission (cardiovascular, respiratory, gastrointestinal and renal/dehydration), nor for patients older than 75 or in other treatment groups. The contribution of each parameter to creatinine increment is presented in Fig 2, where change in 5°C, primary diagnosis of infection and combined use of thiazide + ACE/ARBs affected the increment of serum creatinine when admitted to hospital, more than advanced age, advanced chronic kidney disease at baseline and use of each medication separately.

**Table 4:** Factors associated with change in creatinine levels (creatinine levels at the hospitalization compared to the baseline mean creatinine form the preceding year).

Parameter	Odds Ratio	95% Confidence Interval		
		Lower	Upper	p-value
Intercept	1.009	0.992	1.025	0.280
Change in temperature (5 C°)	1.012	1.009	1.015	<0.001
CKD 3–5	1.000	0.987	1.012	0.944
Age >75	1.008	0.999	1.017	0.063
Any infection	1.045	1.034	1.056	<0.001
ACE/ARBs	1.008	0.997	1.018	0.143
Thiazide	1.016	1.000	1.032	0.047
Thiazide and ACE/ARBs	1.020	1.006	1.034	0.004

ACE/ARBs—angiotensin-converting-enzyme inhibitor or angiotensin receptor blockers

## Discussion

The principal finding of our study is that higher daily temperature is associated with negative effect on kidney function in the elderly population treated with anti-hypertensive medications. This observation was particularly noticeable in patients treated with both thiazides and ACE/ARBs, advanced age above 75 and when the hospitalization index was due to acute infection. Yet, the overall magnitude of the effect appears to have a low clinical significance in the majority of the subjects, e.g increment of 5°C in the ambient temperature was translated into 3.4% increase in the serum creatinine within the dual treatment group.

In the present study, we analyzed an association between ambient temperature and creatinine increment, whereas previous studies examined only sporadic climate effects, e.g. during heat waves (such as August 2003 in France and summer 2009 in Australia)[16] [17]. Frequently the environmental exposure is investigated in conjunction with mortality and hospital admissions. It is well documented that fragile elderly people are more likely to be vulnerable during heat waves to mortality. Several factors such as home nursing residence, pre-existing medical conditions, advanced age (above 80), poor cognitive function and socioeconomic deprivation were shown to be associated with increased risk [18–20]. Furthermore, in a systematic review of the effect of extreme heat and lack of proper environmental adaption in Australia, 11 of the selected 13 studies were focused on mortality [21]. Another systematic review included 33 studies of morbidity during heat waves and demonstrated that the majority were focused on mortality and hospital admission, but only rarely on specific renal damage, possibly due to difficulties in isolating the impact of temperature variation on specific targeted organs [22]. As our data suggest, a comprehensive approach must be considered, taking into account not only the impact of high temperature on specific peaks, but also at continuous daily temperature changes, advanced age, diagnosis of hospital admission and the effect of specific medications. In this regard, elderly patients are more vulnerable to dehydration and to insensible loss, especially during periods of high temperatures [23].

The mechanism of creatinine increase during exposure to the high ambient temperature described in this study may be related to reduced kidney perfusion. AKI has been reported among 5–7% of hospitalized patients, and despite improved awareness it still carries a high mortality risk among the older and sicker patients [24]. These rates may be increasing partially due to predisposing anti-hypertensive medications with hemodynamic effects and invasive diagnostic procedures [25,26]. Medications such as thiazides and ACE /ARBs can facilitate AKI via volume depletion, decreased renal perfusion and a blunted response of the renin-angiotensin system in predisposed elderly patients [27].

In contrast to other anti-hypertensive medications, continuous utilization of thiazide and ACE-inhibitors/ARBs may lead to increase in serum creatinine, and predispose to AKI, especially in persons with pre-existing renal impairment [28–30]. Elderly patients are more vulnerable to dehydration and insensible loss, especially during high temperatures periods [23]. The aging kidney is characterized by structural and functional decompensation [31]. Nevertheless, the aging kidneys are still capable of some compensatory adaptation to climate changes, even in hypertensive elderly patients treated with medications that can impair normal renal function. This hypothesis can explain the increase in creatinine incline, statistically significant yet probably less clinically relevant in a large fraction of our cohort.

Prediction models for AKI are of growing interest. Relevant predicting factors may include age, volemic state, underlying kidney function, diabetes mellitus and CHF, all proven to be associated with future AKI. The additive effects of those factors have been partially tested in previous studies [10,32,33]. In the current study we further expand this concept by showing a novel effect of high temperature on creatinine increment in addition to the age and medications.

We acknowledge several data related limitations. First, this study is of an observational nature and cannot establish causality between temperature changes and renal function. Second, due to the observational settings with data obtained from the health information exchange systems, we could not establish the primary indications to initiate each treatment. The lack of treatment indications bares some major implications on our findings since different indications may lead to different outcomes (for instance the use of ACE-inhibitors/ARBs for hypertension in diabetic versus heart failure patients). Third, we have not assessed the effect of loop diuretics or mineralocorticoid receptor antagonist, since these medications are frequently used to treat heart failure rather than hypertension. Additionally, we have not included other medications that induce excessive fluid loss (e.g. laxatives (or medications that can impair free water clearance by inducing SIADH (e.g. tricyclic antidepressants, selective serotonin reuptake inhibitors and anti psychotics). Fourth, inherently to the ecological type of study we could not assess the personal exposure of the patients to heat or the individual time spent outdoors. Fifth, the results obtained from this study may not be equally applied to patients with different types of renal disorders. Moreover, creatinie measurements prior to hospitalization were available for only 61.5% of the patients. This subgroup showed statistically but not clinically significant differences in baseline characteristics (sicker and older) compared to those without prior creatinine measurement. Sixth, in addition to ambient effect on the dual treatment groups, similar effect was observed on the no-treatment group. This finding may be due to poor medical condition of this group which may also alter medications use, as demonstrated by higher rate of patients with dementia and cancer. Finally, wide use of air-conditioning could minimize the heat exposure in our cohort, and locations of the patients in the preceding days could alter body acclimatization. Of special note, our *statistically* significant data may provide a proof of principle rather than individually *clinically* significant scenarios for a given patient.

## Conclusions

We demonstrated a statistically significant association between ambient temperature and increase in creatinine in elderly patients taking both thiazide and ACE-inhibitors/ARBs. After validating our results in future research, it may be possible to acknowledge that “real life” kidney function has better plasticity than assumed before, enabling more aggressive anti-hypertensive treatment and decreasing co-morbidity complications in the elderly population.

## Supporting Information

S1 Fig Creatinine change (%) between admission and mean creatinine in the previous year, within each season and treatment group. Based on Alpert P et al, *Int J Climatol*. 2004;24(12):1013–1021. ACE/ARB—ang iotensin-converting-enzyme inhibitor or angiotensin receptor blockers.
(TIF)

S1 Table Effect of daily temperature on developing acute kidney injure by medications group. (DOCX)
