# Supplementary figures and images for: The Combined Effect of High Ambient Temperature and Antihypertensive Treatment on Renal Function in Hospitalized Elderly Patients
Source: PLoS One. 2016 Dec 19;11(12):e0168504. doi: 10.1371/journal.pone.0168504 (PMC5167394; doi:10.1371/journal.pone.0168504)

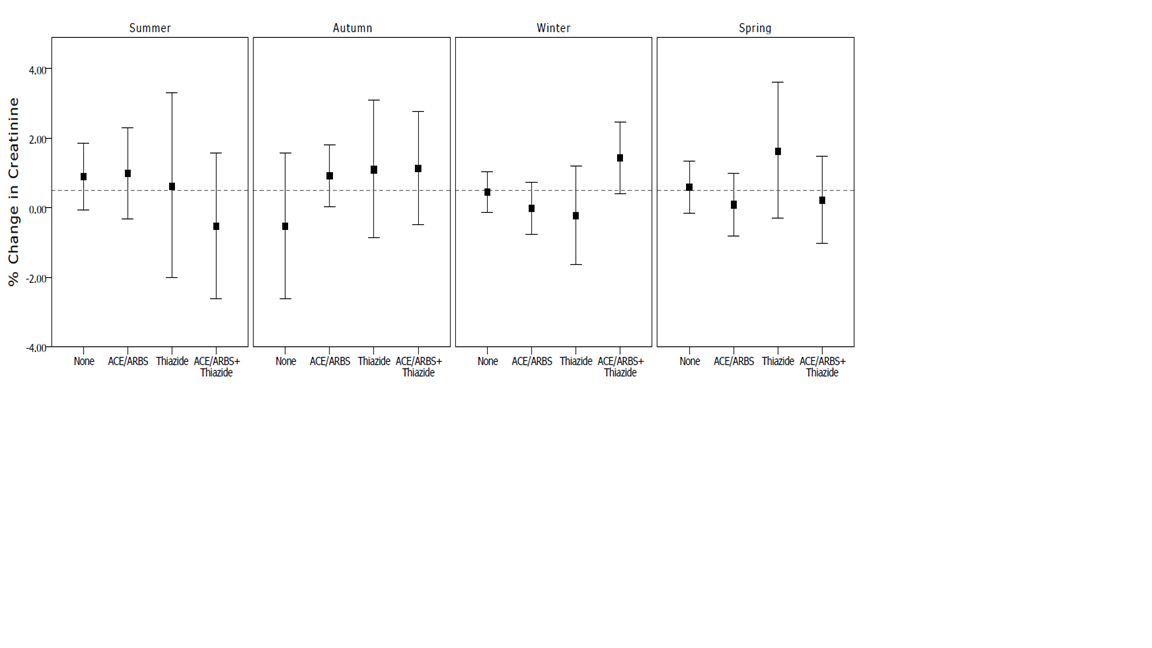

Supplement: S1 Fig — Based on Alpert P et al, Int J Climatol. 2004;24(12):1013–1021. ACE/ARB—ang iotensin-converting-enzyme inhibitor or angiotensin receptor blockers. (TIF) [file pone.0168504.s001.tif]
