# Supplementary material for: The Combined Effect of High Ambient Temperature and Antihypertensive Treatment on Renal Function in Hospitalized Elderly Patients
Source: PLoS One. 2016 Dec 19;11(12):e0168504. doi: 10.1371/journal.pone.0168504 (PMC5167394; doi:10.1371/journal.pone.0168504)
Supplement: S1 Table — (DOCX) [file pone.0168504.s002.docx]

**S1 Table1.** Effect of daily temperature on developing acute kidney injure by medications group

|  | P value | O.R | 95% C.I |
| --- | --- | --- | --- |
| Age | <0.001 | 1.016 | 1.009-1.023 |
| Female | <0.001 | 0.802 | 0.729-0.883 |
| MAP | <0.001 | 0.992 | 0.989-0.995 |
| CHF | <0.001 | 1.978 | 1.688-2.317 |
| PVD | 0.005 | 1.458 | 1.119-1.900 |
| CVA | 0.015 | 1.230 | 1.041-1.455 |
| Diabetes | <0.001 | 1.376 | 1.246-1.519 |
| Liver disease | 0.513 | 1.149 | 0.757-1.745 |
| Dementia | 0.112 | 1.211 | 0.956-1.532 |
| Change in temperature (5 C°) | <0.001 | 1.098 | 1.066-1.130 |
| ACE/ARBS* | 0.113 | 1.100 | 0.978-1.238 |
| Thiazide* | <0.001 | 1.633 | 1.401-1.904 |
| Thiazide and ACE/ARBS* | <0.001 | 1.768 | 1.547-2.021 |

MAP – mean arterial blood pressure, CHF – congestive heart failure, PVD – peripheral vascular disease, CVA – cerebrovascular accident

*no treatment is reference group
